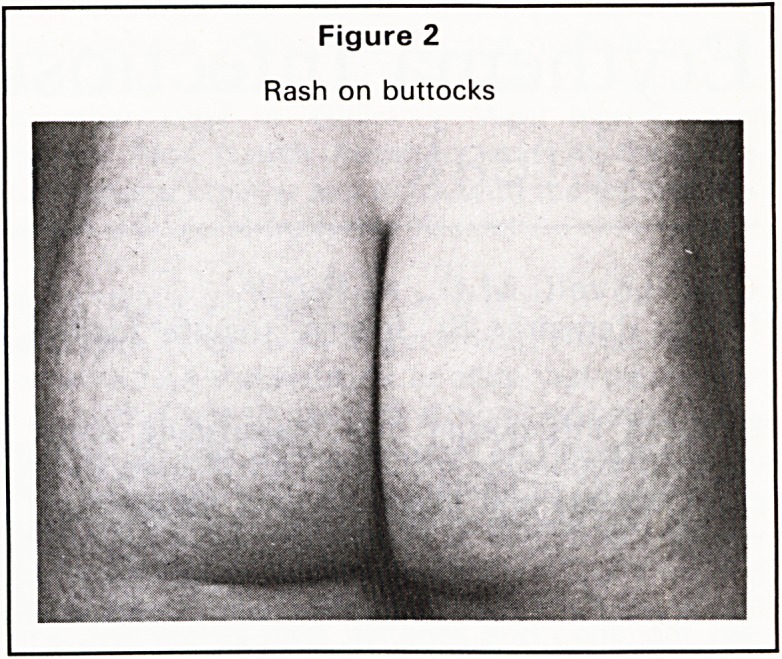# Erythema Infectiosum—A Bristol Outbreak

**Published:** 1985-04

**Authors:** C. R. Lovell

**Affiliations:** Senior Registrar, St. John's Hospital for the Diseases of the Skin, London


### Erythema Infectiosum—A Bristol Outbreak

**C. R. Lovell, M.D., M.R.C.P.**
Senior Registrar, St. John's Hospital for the Diseases of the Skin, London*

Erythema infectiosum may masquerade under a variety of synonyms, including Fifth disease, slapped cheek disease and megalerythema epidemicum. It was first described as a 'peculiarly localised rubella' in Germany and the term 'erythema infectiosum' was first coined in 1896. Since these early descriptions, outbreaks of the disease have been reported from several countries in continental Europe, Japan, Australia and the United States (eg, Auriemma 1954). Reports from the British literature have been scanty. Watts (1974) described two cases and Medhurst (1975) a further six. The first description of an outbreak (of fifty-eight cases) was by Cramp and Armstrong (1976) in North Devon. In 1979–80 we observed a major outbreak of the disease in the Bristol area and presented our findings at a meeting of the British Association of Dermatologists (Lovell et al. 1980). Further outbreaks have occurred in Britain since that time.

Because of the apparently recent arrival of the disease in Britain, some dermatologists and many general practitioners and paediatricians are probably not familiar with it. For this reason, we give a summary of our findings in the Bristol outbreak.

First, two of the synonyms for the diagnosis need some explanation. The term 'slapped cheek syndrome' refers to the transient bright facial erythema observed in children with this condition. The term 'fifth disease' has rather more dubious origins (BMJ leader 1974). There are in fact six conditions in all, listed in the following table.

| | |
|---|---|
| First: | Scarlet fever (Scarlatina) |
| Second: | German measles (Rubella) |
| Third: | Measles (Morbilli) |
| Fourth: | 'Filatow-Dukes' disease, comprising strawberry tongue, albuminuria and desquamation, probably representing a form of scarlatina (BMJ 1974) |
| Fifth: | *Erythema infectiosum* |
| Sixth: | Exanthem subitum (roseola infantum) |

Since the condition occurs in outbreaks in well-demarcated geographical areas, it is highly probable that it is infective in origin, although until recently, attempts to find a viral cause have met with failure.

* Former Registrar in Dermatology, Bristol Royal Infirmary.

With the collaboration of general practitioners in the Bristol area, we were able to examine thirty-four patients with the condition, most of them children. Throat swabs and rectal swabs or stool samples were sent for virology in each patient. Virology samples were also received from a further eighty-three patients seen by their general practitioners. Sixty-two patients were seen in August (1979), twenty-two in September and the remainder scattered over the subsequent months. Seventy-three of the patients were aged under ten years and only nineteen were aged above twenty, nearly all of these being parents of affected children.

The clinical features of the eruption are remarkably consistent within individuals. In children the prodromal illness is absent or very mild, usually consisting of a slight sore throat, low-grade fever or anorexia. One to two days later the 'slapped cheeks' erythema develops and usually only lasts a few hours. The erythema is reticular with a discrete and often irregular edge. It spares the mouth and in fact there is often circumoral pallor. In three children the facial erythema persisted for a few days mimicking the 'butterfly rash' sometimes seen in acute lupus erythematosus. Two to three days later, a blotchy reticulate rash appears on the proximal limbs, particularly the thighs and buttocks (Figures 1 and 2). Over the ensuing days the eruption spreads distally to the forearms and lower legs and disappears within four to five days. The whole eruption generally lasts one week to ten days. Other features include posterior cervical lymphadenopathy but occipital lymphadenopathy was not noticed in any of the children. Physical examination was otherwise normal and all our children were apyrexial. In a few children recurrence of the erythema occurred even up to several weeks later, particularly after vigorous rubbing or after a hot bath.

Differentail diagnosis of the condition is usually not difficult. The characteristic sequence of events in the eruption and the reticulate appearance, together with absence of occipital lymphadenopathy, helps distinguish it from rubella. The children appear too well for scarlet fever which less commonly involves the face. Clinicians with a taste for the esoteric will doubtless consider Chikungunya fever, an arbovirus infection which also leads to facial flushing and a

**Figure 1**

Erythema infectiosum. Blotchy reticulate rash on thighs

**Figure 2**

Rash on buttocks morbilliform rash. This disorder is mostly found in tropical and Southern Africa and the primary host is probably the baboon. (The few baboons permanently resident in Bristol appeared to be well at the time of the outbreak.)

The morphology of the eruption was similar in adults, although generally much less characteristic in appearance and distribution, many parents of affected children having a non-specific morbilliform rash. A few complications were noted in adults. One mother had an acute but transient synovitis of the small joints. Interestingly she gave a family history of Crohn's disease and a history of intermittent diarrhoea. She was found to possess the histocompatibility antigen HLA B27 and it is probable that the infective agent was the trigger for arthritis associated with this tissue-type antigen. Previous reported complications in adults include transient leucopenia, a common consequence of viral infection.

There is considerable clinical evidence to suggest that erythema infectiosum is an epidemic viral infection; it is seasonal, spreads rapidly in closed communities and mainly affects children in their early school years, (BMJ 1984). However, as in previous series, virological studies in our patients were inconclusive. Viruses were isolated in only four patients and included two Adenovirus (Type II and Type VII), Coxsackie A9 and Echo 7. Thus we were unable to identify the pathogenic organism.

Since our study, a more recent report (Anderson and Jones 1983) has indicated that the human parvovirus B19 is the causative agent by the detection of immunoglobulin M antibody in sera from British and foreign outbreaks.

The presence of the parvovirus has not yet been demonstrated, probably because serum is not taken during the incubation period. Parvoviruses were isolated initially in the serum of healthy blood donors and are found in about a third of normal adults (Cossart et al, 1975).

Parvoviruses inhibit erythropoiesis and the human parvovirus is a cause of aplastic crisis in sickle cell anemia (Pattison et al, 1981). It appears likely that erythema infectiosum is a specific, although relatively trivial, manifestation of parvovirus infection.

**ACKNOWLEDGEMENTS**

I am grateful to Drs Harman, Peachey, Burton and Warin for allowing me to report their patients, Dr. Suzanne Evans for virological studies, Dr. J. Wright who drew our attention to the outbreak, and the many General Practitioners who collaborated by sending their cases for investigation.

### REFERENCES

ANDERSON, M. J., JONES, S. E., FISHER-HOCH, S. P. et al. (1983) Human parvovirus, the cause of erythema infectiosum (fifth disease)? *Lancet* **1**, 1378.

AURIEMMA, H. A. (1954) Erythema infectiosum: Report on a familial outbreak. *Amer. J. of Pub. Health* **44**, 1450–1454.

BRITISH MEDICAL JOURNAL (1974) Fourth, fifth and sixth (Leader). *Br. Med. J.* **4**, 429.

BRITISH MEDICAL JOURNAL (1984) The 80th year of fifth disease (Leader). *Br. Med. J.* **284**, 338–9.

COSSART, Y. E., FIELD, A. M., CANT, B., WIDDOWS, D. (1975) Parvovirus – like particles in human sera. *Lancet* **1**, 72–3.

CRAMP, H. E. and ARMSTRONG, B. D. J. (1976) Erythema infectiosum: an outbreak of 'slapped cheek' disease in north Devon. *Br. Med. J.* **1**, 885–6.

LOVELL, C. R., WRIGHT, J. C., BOSS, J. M., WARIN, R. P., HARMAN, R. R. M., PEACHEY, R. D. G., BURTON, J. L. and CLARKE, S. K. R. (1980) An epidemic of erythema infectiosum *Brit. J. Derm.* **103**, 24 (meeting abstract).

MEDHURST, A. W. (1975) Erythema infectiosum. *Br. Med. J.* **1**, 394.

PATTISON, J. R., JONES, S. E., HODGSON, J. et al. (1981) Parvovirus infections and hypoplastic crisis in sickle cell anemia. *Lancet* **1**, 664–5.

WATTS, C. A. (1974) Erythema infectiosum. *Br. Med. J.* **4**, 466–467.

## Figures and Tables

**Figure 1 f1:**
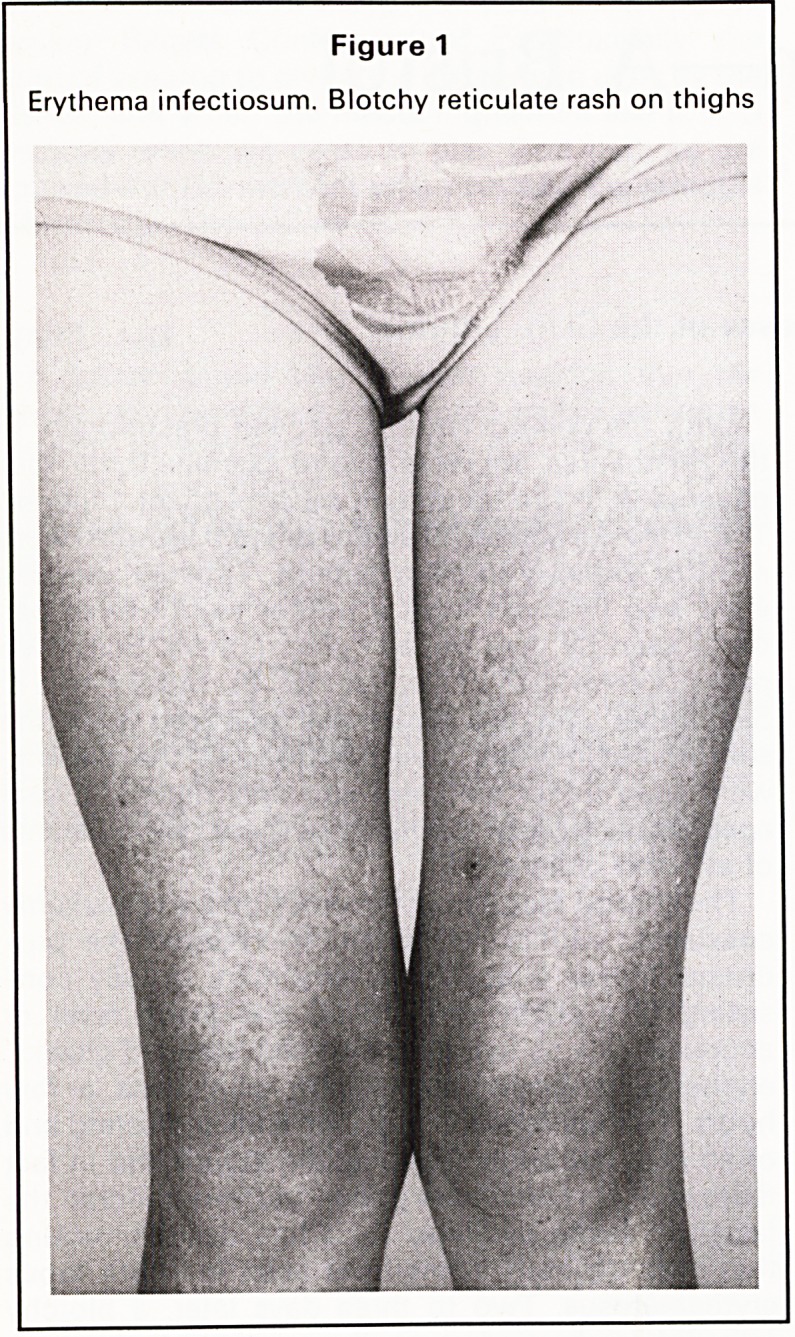


**Figure 2 f2:**